# Efficacy of Plasma Rich in Growth Factors (PRGF) in Stage 1 Neurotrophic Keratitis

**DOI:** 10.21203/rs.3.rs-3040369/v1

**Published:** 2023-06-30

**Authors:** Seyyedehfatemeh Ghalibafan, Kwaku Osei, Guillermo Amescua, Alfonso Sabater

**Affiliations:** University of Miami Miller School of Medicine; University of Miami Miller School of Medicine; Bascom Palmer Eye institute, University of Miami, Miller School of Medicine; Bascom Palmer Eye Institute

**Keywords:** Neurotrophic keratitis, Plasma rich in growth factors, Ocular surface disease

## Abstract

**Background/Aims:**

Neurotrophic keratitis (NK) is a neurodegenerative disease that can lead to corneal hypoesthesia, decreased tear production, and epitheliopathy. Based on the severity of ocular surface damage, NK is classified into 3 stages. Stage 1 NK is characterized by superficial punctate keratopathy, tear film instability, and reduced corneal sensation. The therapeutic efficacy of PRGF eye drops for NK stages 2 and 3 has been previously reported. In this study, we evaluated the efficacy and safety of autologous PRGF eye drops in improving corneal sensitivity and other ocular surface clinical signs in patients with stage 1 NK.

**Methods:**

Retrospective chart review

**Results:**

26 eyes of 15 stage 1 NK patients (seven males, eight females), aged 76.3 ± 12.1 years, were included in the study. The mean treatment duration was 2 ± 1.8 months. With PRGF treatment, corneal sensitivity increased from 2.8 to 4.5 cm in 53.8% (14/26) (p < 0.01), TBUT increased from 3.6 to 5.0 s in 69.2% (18/26) (p < 0.01), and Schirmer score increased from 13.7 to 16.8 mm in 80.7% (21/26) of treated eyes (p < 0.01). Similarly, an improvement in corneal staining (punctate epithelial erosions) and MMP-9 levels was seen in 80.7% (n = 21) and 65.4% (n = 17) of treated eyes, respectively. BCVA improvement was seen in 26.9% of treated eyes (n = 7).

**Conclusions:**

This study demonstrates the effective role of PRGF therapy in recovering corneal sensation and tear film function and in the healing of corneal erosions in stage 1 NK patients.

## Introduction

Classified as an orphan disease (ORPHA137596), neurotrophic keratitis (NK) is a chronic degenerative corneal disease with an incompletely understood pathophysiology and a broad clinical spectrum (1–3). Its prevalence had been estimated to be 5–11 cases per 10,000 overall, but the recent Intelligent Research in Sight (IRIS) Registry Analysis by Bian et al. reported the 6-year prevalence of NK as 21.34 cases per 100,000 patients in the United States (4–7).

The ophthalmic branch of the Trigeminal nerve (Cranial Nerve V) is the main nerve that supplies cornea innervation and provides pivotal trophic factors and nutrition for the maintenance of ocular surface health, corneal integrity, and function (8–10). This nerve malfunction potentially results in neurotrophic phenomena accompanied by many ocular surface diseases that are classified as neurotrophic keratopathies. They are mainly associated with corneal sensitivity reduction, epithelial and stroma alteration, ulceration, melting, and perforation (11–13).

Moreover, trigeminal nerve damage can affect tear film production through a loss of functioning in the protective sensory-mediated reflexes such as the tear gland reflex and blinking. The intact interaction between tear film, epithelium layer, and sensory innervation is essential for the stability of the epithelium (10, 14–17). NK can cause temporary or even permanent visual dysfunction as a result of the inability to resynthesize the corneal epithelium, the enzymatic deterioration of stroma, and the decrease of tear production. Therefore, early detection, complete therapy, and strenuous follow-up of NK patients are crucial (5, 18–22).

Common causative factors of NK are infectious keratitis (herpes zoster and herpes simplex), previous corneal surgery or trauma, corneal dystrophy, contact lens abuse, chemical burn, neurosurgical procedures with trigeminal nerve transection, antipsychotic treatments, and systemic diseases like diabetes mellitus, multiple sclerosis, Sjogren’s syndrome, and rosacea (2, 4, 23–27). Additionally, some genetic syndromes such as familial dysautonomia, Goldenhar-Gorlin syndrome, familial corneal hypoesthesia, and congenital insensitivity to pain with anhidrosis have been demonstrated to underlie NK (28–31).

Although corneal hypoesthesia is the main clinical manifestation of NK, patients usually do not have any ocular surface discomfort in the primary stages which leads to delayed detection. Clinical signs and symptoms in NK patients are mostly related to tear film abnormality and dry eye symptoms (short tear-break-up time (TBUT), decreased Schirmer test), reduced blink rate, decreased corneal sensitivity, punctate epithelial erosions (PEE), and cloudy epithelium on slit-lamp examination. Blurred vision and even vision loss are serious consequences of NK, caused by tear film abnormalities, epithelial erosions and/or defects, corneal stroma scarring, or swelling (9, 12, 32).

Based on the severity of damage on the ocular surface and corneal sensation, NK was first arranged into three stages according to the Mackie classification in 1995 (33). The most recent classification for NK was proposed by Mastropasqua *et al*. and Dua *et al*. using *in vivo* confocal microscopy (IVCM) and anterior segment optical coherence tomography (AS-OCT) to estimate the severity of subbasal nerve fiber damage and stromal ulceration extension (8, 34). Stage 1 NK has a higher prevalence than stages 2 and 3 and is characterized by mild epithelial and tear film alterations, superficial punctate keratopathy, corneal edema, and reduced or absent corneal sensation in one or more quadrants of the cornea. Stage 2 NK is associated with moderate epithelial defect without stromal defect, while stage 3 NK presents with severe corneal ulcers, stromal melting, and perforation (1, 8, 9, 35).

The therapeutic approach in NK patients is multimodal and aimed at the prevention of disease progression, promotion of epithelial healing, and treatment of the underlying denervation and concomitant ocular diseases such as dry eye and limbal stem cell deficiency (18, 36). Common treatments for NK patients vary based on the NK stage and include topical therapies such as cenegermin (recombinant nerve growth factor), preservative-free artificial tears, autologous serum or plasma tears, and invasive surgical modalities such as corneal neurotization and transplantation, tarsorrhaphy, amniotic membrane placement, and scleral contact lenses (23, 37–41).

Nowadays, outstanding clinical outcomes are reported by using autologous blood derivates such as autologous serum (AS) eye drops and plasma rich in growth factors (PRGF-Endoret) eye drops as regenerative treatments for multiple ocular surface disorders such as those affecting patients with glaucoma and post-photorefractive keratectomy (PRK), corneal epithelial defects and ulcers in NK, ocular graft versus host disease, cicatrizing conjunctivitis, and dry eye syndrome (19, 42–45). The autologous growth factors, proteins, and biologically active constituents with antimicrobial and anti-inflammatory nature found in these drops have been present in the tear fluid and enable its vital hemostasis and nurturing of the cornea (46).

PRGF eye drops are a promising therapy made by a standardized process using a special kit (PRGF-Endoret^®^, BTI-Biotechnology Institute, Vitoria, Spain). Through the process, eye drop bottles are aseptically filled with the patient’s blood as a pool of biologically active proteins that facilitate epithelial cell regeneration (12, 47). Although some investigations report the curative effects of PRGF in reversing the course of advanced NK (stage 2 and stage 3), there is still sparse data on the clinical features, treatment responses, and outcomes of patients with stage 1 NK treated with PRGF-Endoret^®^ eye drops (12). In the present study, we evaluated the efficacy and safety of PRGF eye drops in the recovery of corneal sensitivity and tear film function as well as the clinical signs of ocular surface improvement in patients with stage 1 NK.

## Patients and methods

This was a retrospective chart review of stage 1 NK patients who visited cornea service at the Bascom Palmer Eye Institute between November 2021 and December 2022. Patients were instructed to use PRGF eye drops 4–6 times daily for at least two months. Preservative-free artificial tear eye drops were offered to all patients as needed between PRGF applications, beginning with the initial visit. PRGF eye drops were prepared by the Florida Lions Eye Bank (Miami, FL) using the Endoret^®^ PRGF kit (BTI, Vitoria-Gasteiz, Spain). Demographic data and ocular surface clinical parameters were recorded at the baseline visit and after treatment with PRGF.

### Main outcomes:

The main outcomes included corneal sensitivity measured with the handheld Luneau Cochet-Bonnet esthesiometer (CBE, Luneau ophthalmologia, Chartes Cedex, France), tear film break-up time (TBUT), Schirmer’s score, corneal fluorescein staining (0–5 Oxford scale), matrix metalloproteinase 9 level (MMP-9, InflammaDry^®^, Quidel, San Diego, CA, USA) and best-corrected visual acuity (BCVA). All patients underwent slit lamp and Keratograph 5M (Oculus, Germany) using fluorescein staining to assess the ocular surface changes in the treatment course. Complications were considered as a new development or a progression of epithelial defects. This study was approved by the University of Miami Institutional Review Board. The protocols and methods used also complied with the standards set forth by the Declaration of Helsinki.

### Data analysis:

Data captured were analyzed in GraphPad Prism (Version 8). Differences in corneal sensitivity, tear breakup time, and Schirmer scores measured before and after PRGF treatment were analyzed using the Wilcoxon matched-pairs signed rank test, with p-value < 0.05 denoting statistical significance.

## Results

A total of 26 eyes from 15 patients with NK were included in the study. Eight patients were women (53%) and seven were men (47%). The presentation was unilateral in 26.7% and bilateral in 73.3%. The mean age was 76.3 ± 12.1 years (range: 48–88 years) and the mean treatment duration was 2 ± 1.8 months. The most frequent causes of stage 1 NK in our patients were iatrogenic (glaucoma medications) in 26.7% (4/15), dry eye syndrome in 26.7% (4/15), and herpes simplex virus in 20% (3/15) of cases. Previous ocular surgeries were seen in 40% (6/15) of patients. Patients’ demographic characteristics are summarized in Table 1.

**Figure 1:**
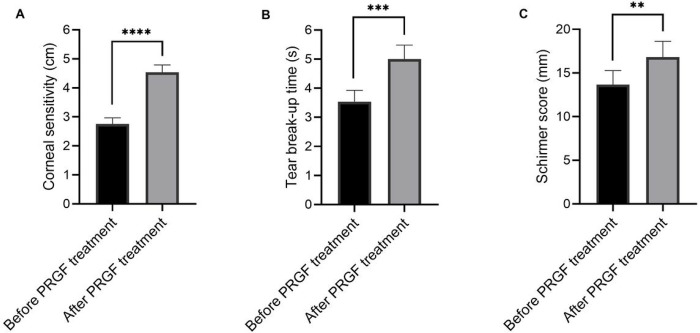
Changes in corneal sensitivity and tear film parameters after treatment with PRGF eye drops.

With PRGF treatment, corneal sensitivity increased from 2.8 cm to 4.5 cm in 53.8% (14/26) (Fig. 1A, p < 0.0001), tear breakup time increased from 3.6 s to 5.0 s in 69.2% (18/26) (Fig. 1B, p = 0.0007) and Schirmer score increased from 13.7 mm to 16.8 mm in 80.7% (21/26) of treated eyes (Fig. 1C, p = 0.0029). Similarly, an improvement in corneal staining (PEE) and MMP-9 levels were seen in 80.7% (n = 21) and 65.4% (n = 17) of treated eyes, respectively. BCVA improvement was seen in 26.9% of treated eyes (n = 7). There were no adverse events reported during the treatment course.

## Discussion

NK is a rare degenerative disease of the corneal subbasal nerve plexus with an insidious evolution (48). Its management and accurate diagnosis are challenging due to the diverse underlying ocular and systemic diseases such as dry eye, tear film alteration, limbal stem cell dysfunction, corneal dystrophies, chronic use of topical medications, herpetic infection, and diabetes mellitus (49–53). Ocular and neuro surgeries that affect the cornea such as cataract surgery, refractive surgery, keratoplasty, acoustic neuroma, trigeminal neuralgia, and neoplasms result clinically in NK by impairing trigeminal innervation on the surgical area (54–56). Six (40%) of our patients had previous ocular surgery with the majority having cataract surgery. The antecedents that occurred most frequently among them were glaucoma medications, dry eye syndrome, and herpetic NK in 26.7%, 26.7%, and 20% of cases, respectively. NK prevalence had been reported as 5–11 cases/10,000 population; however, recent studies have demonstrated that this approximation seems to underestimate the real numbers (3, 4, 57).

The main strategy in stage 1 NK treatment is to improve epithelial quality and stability, accelerate corneal healing, and prevent stromal involvement (9). NK therapy includes a diverse spectrum such as nerve growth factor (NGF) eye drops, neuropeptides (substance P), surgical therapy, and autologous blood-based eye drops (48, 58, 59). OXERVATE^®^ (cenegermin-bkbj), an FDA-approved topical recombinant human nerve growth factor, was reported as an efficient therapy in moderate to severe NK that can lead to significant improvement in corneal sensation and complete resolution of epithelial defect (60–62). Bandage contact lenses (BCLs) are another therapy option for NK patients that provide corneal epithelium protection; they are widely used in some conditions like exposure keratopathy, limbal stem cell deficiency (LSCD), and severe dryness (60, 63). Unfortunately, despite the curative effects of autologous serum and cord blood eye drops in modulating NK progress, they have toxic pro-inflammatory components that limit their usage (64–66).

A new regenerative treatment, PRGF–Endoret^®^ eye drops play a determinantal role in promoting corneal healing of diverse ophthalmic pathologies such as NK and dry eye syndrome by having a higher concentration of growth factors and antimicrobial agents with reduced pro-inflammatory cytokines than other blood-based treatments (47, 67–69). To the best of our knowledge, this study is the first report on the safety and efficacy of PRGF treatment in stage 1 NK patients.

Several studies have demonstrated that excessive inflammation can cause corneal innervation damage, especially in NK patients, so modulation of the inflammatory pathway has an essential role in corneal defect/ulcer healing (12, 70–73). In our studies, reduced inflammation (MMP-9 level, Inflammadry^®^) was seen in 65.4% (17/26) of eyes after PRGF treatment eye drops. In addition to matrix metalloproteinase inhibitors nature, PRGF therapy has been shown to have antinociceptive properties that inhibit the NF-κB pathway and reduce abnormal sensation in NK patients (74, 75). The most frequent clinical characteristics of our patients were blurry vision, dry eye, and foreign body sensation in 60% (9/15), 86.7% (13/15), and 53.3% (8/15) of cases, respectively; those clinical characteristics successfully resolved in all of our cases after treatment. Those data are in line with those reported in other literature (4, 54, 76).

Sanchez et al. demonstrated that 99.4% of the patients with stage 2 and stage 3 NK had complete resolution of corneal defects and ulcers within three months after treatment with PRGF- eye drops. They also reported significant improvement in BCVA in 52.8% of patients (12). Likewise, our study demonstrated healing of corneal epithelial erosions and improved visual acuity in 80.7% (21/26) and 26.9% (7/26) of treated eyes, respectively, after an average of two months of therapy with PRGF. Similar to other series, there were no serious adverse effects among our patients, which emphasizes the efficacy and safety of PRGF as an alternative therapy for the treatment of both initial and advanced stages of NK (19). In studies by *Merayo-Lloves* and *Kim*, PRGF eye drops were shown to successfully improve BCVA and complete corneal re-epithelialization in patients with infectious keratitis and refractory ocular surface disorders who were non-responders to autologous serum tears or cyclosporine (77, 78).

Decreased corneal sensitivity in NK patients can reduce tear production, which triggers a vicious circle that ultimately deteriorates the corneal epithelium through drying and breaking down (9, 79). In a study by *Roszkowska et al*., corneal sensitivity was recovered successfully by a significant increment in all NK patients (stage 2 and stage 3) after cenegermin therapy. The results from the present study show that tear film production and corneal sensitivity improved significantly (P-value < 0.01) in 80.7% (21/26) and 53.8% (14/26) of treated eyes, respectively, with an eight-week course of PRGF-eye drops.

In conclusion, we have shown the effective role of PRGF therapy in improving ocular surface parameters in stage 1 NK patients.

## Limitations:

This was a retrospective, non-comparative nature review with limitations such as being unable to control other potential confounders.

## Figures and Tables

**Figure 2:**
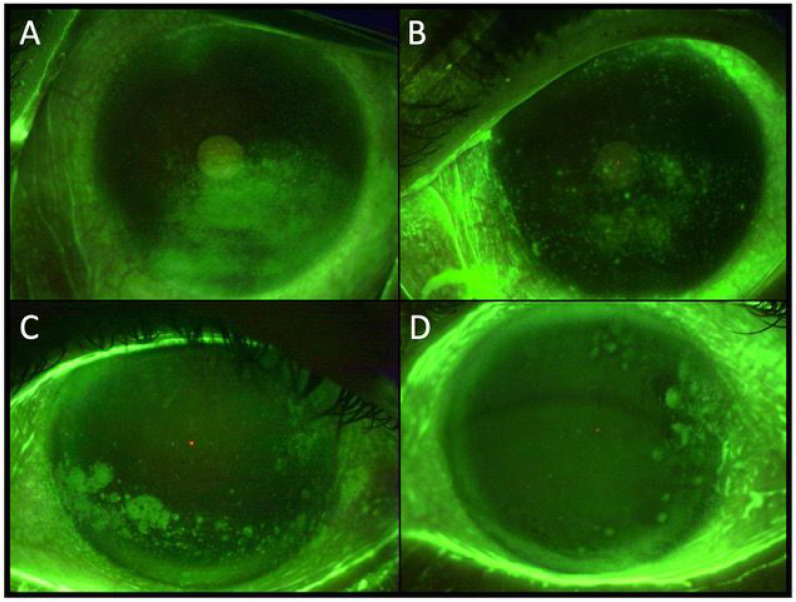
**Figure 2 and Figure 3:** Represent the initial and final status of two cases with stage 1 NK after 8.3 and 8.5 weeks of treatment with PRGF eye drops. a: basal status, b: final status of patient after PRGF treatment.

**Table 1:** Demographic characteristics of Satge 1 NK patients

Patient	Gender	Age range (years)	Eye involvement	Etiology of NK	Contact lens use	Previous ocular surgery
1	Female	70	Both	Iatrogenic (Glaucoma drops)	+	−
2	Female	80	left	Herpetic NK	+	−
3	Female	60	left	Herpetic NK	−	Cataract
4	Male	80	Both	Dry eye syndrome	−	−
5	Female	70	Both	Rosacea	−	−
6	Male	90	Both	Fuch’s endothelial dystrophy	−	Cataract + Fuch’s surgery
7	Female	90	Both	Herpetic NK	−	−
8	Female	70	Both	Dry eye syndrome	−	Cataract + Pterygium
9	Female	90	Both	Dry eye syndrome	+	−
10	Male	70	left	Pseudophakic bullous keratopathy + Iatrogenic (Glaucoma drops)	−	Multiple glaucoma surgeries
11	Male	90	Both	Iatrogenic (Glaucoma drops)	−	−
12	Female	80	Both	Dry eye syndrome	−	−
13	Male	50	left	Neurosergical cause (meningioma)	+	−
14	Male	90	Both	Previous vitrectomy + laser retinopexy	−	Radial keratotomy
15	Male	80	Both	Iatrogenic (Glaucoma drops)	−	Cataract
